# p53 autoantibodies in patients with malignant mesothelioma: stability through disease progression

**DOI:** 10.1054/bjoc.2000.1529

**Published:** 2001-01-01

**Authors:** J Creaney, B M McLaren, S Stevenson, A W Musk, N de Klerk, B W S Robinson, R A Lake

**Affiliations:** Western Australian Institute for Medical Research and University Department of Medicine, University of Western Australia, Queen Elizabeth II Medical Centre, 4th Floor, G Block, Nedlands, Perth, 6009, Western Australia

## Abstract

Malignant mesothelioma (MM) generally occurs as a pleural tumour, related to the inhalation of asbestos fibres. It is highly aggressive and largely unresponsive to treatment. The incidence of MM is particularly high in Western Australia because of the extensive blue asbestos mining operations that occurred in the north of the state until 1966. MM is unusual in that mutations in the tumour suppressor gene *p53* are rarely observed, whilst over-expression of p53 protein is common. As the level of antibodies directed against p53 is thought to be of prognostic value in some cancers and as MM is known to be immunogenic, we studied a cohort of Western Australian patients to determine the prevalence of anti-p53 antibodies and their value as diagnostic markers or prognostic indicators. 6/88 (7%) of patients had high titres (>2 SD above the mean of controls) of anti-p53 antibodies. There was no correlation between antibody titre and survival. Although 3/38 (8%) of sera obtained from patients exposed to asbestos but prior to a diagnosis of MM contained antibodies, the same proportion of sera obtained from patients exposed to asbestos but who remained disease free also contained antibodies (2/40; 8%). Sera collected sequentially demonstrated a profound temporal stability in the titre of anti-p53 antibodies in patients with MM throughout the course of their illness. These results show that anti-p53 antibodies are observed only at a low frequency in the sera of MM patients and where they do occur, their elicitation is an early event that may be unrelated to antigen load. The occurrence of anti-p53 antibodies does not serve as either a useful prognostic or diagnostic indicator in MM. © 2001 Cancer Research Campaign http://www.bjcancer.com


*British Journal of Cancer* (2001) **84**(1), 52–56
© 2001 Cancer Research Campaign
doi: 10.1054/ bjoc.2000.1529, available online at http://www.idealibrary.com on IDEAL®

http://www.bjcancer.com

### p53 autoantibodies in patients with malignant mesothelioma: stability through disease progression

**J Creaney, BM McLaren, S Stevenson, AW Musk, N de Klerk, BWS Robinson and RA Lake**

Western Australian Institute for Medical Research and University Department of Medicine, University of Western Australia, Queen Elizabeth II Medical Centre, 4th Floor, G Block, Nedlands, Perth, Western Australia 6009

**Summary** Malignant mesothelioma (MM) generally occurs as a pleural tumour, related to the inhalation of asbestos fibres. It is highly aggressive and largely unresponsive to treatment. The incidence of MM is particularly high in Western Australia because of the extensive blue asbestos mining operations that occurred in the north of the state until 1966. MM is unusual in that mutations in the tumour suppressor gene *p53* are rarely observed, whilst over-expression of p53 protein is common. As the level of antibodies directed against p53 is thought to be of prognostic value in some cancers and as MM is known to be immunogenic, we studied a cohort of Western Australian patients to determine the prevalence of anti-p53 antibodies and their value as diagnostic markers or prognostic indicators. 6/88 (7%) of patients had high titres (>2 SD above the mean of controls) of anti-p53 antibodies. There was no correlation between antibody titre and survival. Although 3/38 (8%) of sera obtained from patients exposed to asbestos but prior to a diagnosis of MM contained antibodies, the same proportion of sera obtained from patients exposed to asbestos but who remained disease free also contained antibodies (2/40; 8%). Sera collected sequentially demonstrated a profound temporal stability in the titre of anti-p53 antibodies in patients with MM throughout the course of their illness. These results show that anti-p53 antibodies are observed only at a low frequency in the sera of MM patients and where they do occur, their elicitation is an early event that may be unrelated to antigen load. The occurrence of anti-p53 antibodies does not serve as either a useful prognostic or diagnostic indicator in MM. © 2001 Cancer Research Campaign http://www.bjcancer.com

Some of the most commonly described genetic mutations in human cancers occur within the *p53* gene. *p53* is a tumour suppressor that functions to protect the cell by regulating cell cycle progression following DNA damage and other stresses (Levine, 1997). Mutations in the gene can increase the half-life of the protein causing a loss of protective function as mutant protein accumulates in the nuclei of tumour cells. The consequences of the overexpression of mutant protein are not fully understood but have been shown, in some instances, to lead to an immune response characterized by the production of anti-p53 auto-antibodies (Angelopoulou et al, 1994; Lubin et al, 1995).

Anti-p53 antibodies have been detected in the serum of patients with a range of cancers. However, not all patients with cancer develop anti-p53 antibodies. The frequency varies depending on the type of assay employed and the patient sampling criteria. Anti-p53 antibodies have been detected in up to 26% of lung, 19% of pancreatic, 17% of bladder, 13% of breast, 26% of colorectal and 46% of ovarian cancers (Lubin et al, 1995; Hammel et al, 1999; Vogl et al, 1999).

A lot of interest has been generated in anti-p53 antibodies in the hope that they may serve as diagnostic markers or prognostic indicators of disease severity. Recent work has shown that breast (Lenner et al, 1999), lung (Lai et al, 1998), gastric (Wu et al, 1999) and urological (Lang et al, 1998) cancer patients with anti-p53 antibodies had a significantly poorer prognosis than p53 antibody-negative patients. However, in other forms of cancer such as head and neck (Gottschlich et al, 1999), non-small cell lung (Mitsudomi et al, 1998), ovarian (Gadducci et al, 1999), and gastric cancer (Nakajima et al, 1999) the presence of antibodies against p53 showed no correlation with patient prognosis. Interestingly, it has been shown for a sub-set of patients with pancreatic cancer (Gansauge et al, 1996) and with hepatocellular cancer (Saffroy et al, 1999) that the presence of p53 antibodies is associated with a relatively ‘good prognosis’. It seems clear that the importance of p53 auto antibodies as a prognostic indicator is likely to be dependent on tumour type.

MM is an incurable cancer associated with exposure to asbestos. MM is characterized by a long latency period and diagnosis at an advanced stage. It is highly resistant to both chemotherapy and radiotherapy, with poor prognosis; the mean survival time following presentation is 9 months (Musk et al, 1992). MM is generally considered to be non-immunogenic or at best partially immunogenic because immunization of mice with irradiated MM cells confers little or no protection to a tumorigenic rechallenge. There is however, good evidence for a weak anti-tumour response in patients. Up to 20% of patients respond to IFN α or GMCSF treatment (Upham et al, 1993; Davidson et al, 1998) and the majority of MM patients exhibit anti-tumour antibody responses. Antibody responses to native tumour antigens can be demonstrated in up to 30% of patients by Western blot (Robinson et al, 1998). Using SEREX, we have shown that private specificities dominate this response and that the complexity of the response increases as the tumour grows, perhaps indicating thresholds that are crossed through increasing antigen concentration (Robinson et al, 2000). In all of these studies, we have restricted our analyses to class-switched IgG isotypes as an indication of T cell recognition of the antigen.

*Received 28 June 2000*
*Revised 12 August 2000*
*Accepted 18 August 2000*

*Correspondence to:* RA Lake

MM rarely, if ever, exhibit mutations in the *p53* gene (Metcalf et al, 1992; Andersson et al, 1995; Lechner et al, 1997; Mor et al, 1997; Lee and Testa, 1999; Mayall et al, 1999), however, the proportion of tumours reported to over-express p53 is relatively high, with frequencies that range from 25% (9 out of 36) (Ramael et al, 1992) to 85.7% (30 out of 35) (Esposito et al, 1997). In normal cells, wild-type p53 is present at low levels because the protein is very rapidly degraded following synthesis (Ashcroft and Vousden, 1999). Mutation of p53 often results in the synthesis of a stabilized protein that has a much longer half-life than that of the wild-type protein, therefore, immunohistochemical techniques are generally employed to detect mutant p53 (Angelopoulou et al, 1994; Lubin et al, 1995). There is, therefore, a discrepancy between findings of wild-type p53 and over-expression of p53 protein in MM, and the reasons for this are not clear. However, the over-expression of p53 in MM may elicit anti-p53 antibodies which may provide an important surrogate marker of disease severity. A recent study showed that MM patients had anti-p53 antibody titres that were not different from controls (Schneider et al, 1999), however the sample size was small ($n$=5), so we examined the prevalence of antibodies to p53 in a larger sample of MM patients seeking to correlate antibody levels with clinical outcome. Early diagnosis of MM may help to improve the poor prognosis if improved treatment can be applied. We, therefore examined anti-p53 antibodies in pre-diagnosis sera to determine if their occurrence might provide an early indication of the disease. Finally, because there are no surrogate markers of disease progression, we examined whether antibody levels paralleled antigen load.

### MATERIALS AND METHODS

### Sera

Blood was obtained from patients presenting at the respiratory clinics at Sir Charles Gairdner Hospital (Perth, Western Australia) and from normal healthy laboratory volunteers. The procedures were approved by the Human Rights Committee of the University of Western Australia and fulfilled NH&MRC Guidelines on Human Experimentation and informed consent was obtained from all patients before enrolment in the study. Clotted blood was centrifuged at 3000 ***g*** for 10 min and serum stored in aliquots at –80˚C before use. Diagnosis of MM was made from history and by radiological examination and confirmed by cytology and immunohistochemistry. The patients (76 male and 12 female) were aged between 39 and 81 years at the time samples were taken (mean 63.4 ± 9.6 years). The normal controls (18 male and 7 female) were aged between 24 and 74 years (mean 45.2 ± 17 years). As part of an independent public health study, sera were collected annually from individuals previously exposed, either occupationally or environmentally, to blue asbestos in the Wittenoom area of Western Australia, some of these individuals went on to develop MM (de Klerk et al, 1998; Musk et al, 1998). A subset of this serum bank was used in the current study, serving as an age-matched, asbestos-exposed control population and as a source of pre-diagnosis sera.

### Cell lines and tissue culture

The human MM cell lines, JU77, LO68, NO36, ONE58 and STY51 were all derived from the pleural fluid of patients with disease (Manning et al, 1991). The human lung adenocarcinoma, A549, and the human colon cancer line HT29 were purchased from the American Type Culture Collection (Manassas, VA). Cells were cultured in RPMI-1640 (Life Technologies, Melbourne, Australia) supplemented with 20 mM HEPES, $5 \times 10^{-5}$ M 2-mercaptoethanol, 10% foetal calf serum (Life Technologies) and incubated at 37˚C in a water-saturated atmosphere of 5% $CO_2$ in air.

### Western blot analysis

Peripheral blood lymphocytes (PBL) were obtained from heparinized venous blood by Ficoll-Paque (Pharmacia, Upsala, Sweden) density gradient centrifugation. Cells from log phase in vitro cultures were harvested by centrifugation. Cell lysates were separated by electrophoresis and blotted onto membranes as previously described (Robinson et al, 1998), with p53 expression examined using a murine anti-p53 antibody (DO-1, Santa Cruz Biotech, CA) at a 1:5000 dilution in PBS. The intensity of signal produced following visualization of bound antibody with chemiluminescence (ECL; Amersham Life Sciences, England) was determined using ImageQuant software (Molecular Dynamics, Sunnyvale, CA).

### ELISA

Wells of a 96-well microtitre plate (COSTAR, Corning) were coated with p53 (Santa Cruz Biotechnology) at 250 ng $ml^{-1}$ in 100 mM $NaHCO_3$, pH 8.2 overnight at 4˚C. Wells were washed three times with PBST, blocked for 1 h at 37˚C with 75 µl $well^{-1}$, PBS, 20% normal horse serum and re-washed. Serum samples were diluted at 1/50 in PBST then applied in duplicate to p53-coated and control (buffer only) plates, a double dilution was performed so that samples were also assessed at a 1/100 dilution. Plates were incubated for 1 h at 37˚C then washed. Bound antibodies were detected following 1 h, 37˚C incubation with alkaline phosphatase conjugated anti-human IgG (Promega) and visualized with 50 µl $well^{-1}$ 1 mg $ml^{-1}$ pNPP (Sigma) in 100 mM $NaHCO_3$, 100 mM $Na_2CO_3$ pH 9.8, 2 mM $MgCl_2$. Absorbance was measured on a SpectrMax 250 plate reader at 405 and 490 nm and presented as L1–L2. Relative absorbance was determined by subtracting values for control plates from p53 plates. The ELISA was highly reproducible and variance between duplicates did not exceed 5%. We were able to demonstrate the specificity of the assay by complete inhibition of binding with recombinant p53 but no inhibition with an unprogrammed bacterial lysate (data not shown).

### RESULTS

### p53 protein expression

Using an antibody that recognizes both mutant and wild-type human p53, p53 was detected in all samples tested by Western blotting. In normal PBL, wild-type p53 was of low abundance (Figure 1). Low p53 levels were also demonstrated in the A549 lung adenocarcinoma cell line, in which the *p53* gene is unmutated. Mutant p53 has a longer half-life than the wild-type protein (Angelopoulou et al, 1994; Lubin et al, 1995) so the presence of high levels of p53 protein may indicate the presence of a mutated *p53* gene (Figure 1; HT29 cells carry a GA mutation at codon 273).

For comparison, one tenth of the amount of HT29 protein was examined compared to the other cell lysates. All of the human MM cell lines examined over-expressed p53 protein compared with PBL (Figure 1). There was variation in the level of p53

© 2001 Cancer Research Campaign 

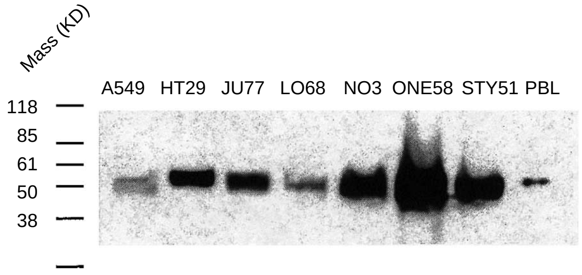

**Figure 1** Expression of p53 protein by normal cells and MM cell lines. Lysates were prepared from the human lung cancer cell line (A549), the colon cancer cell line (HT29), various MM cell lines (JU77, LO68, NO3, ONE58 and STY51) and from normal peripheral blood lymphocytes (PBL). Proteins were separated electrophoretically and blotted onto nitrocellulose. The amount of p53 protein in each lysate was determined by exposing the blot sequentially to a specific monoclonal anti p53 antibody (DO-1), rabbit anti mouse IgG labelled with HRP and to the ECL development reagent. Note: HT29 lysate was diluted 1/10

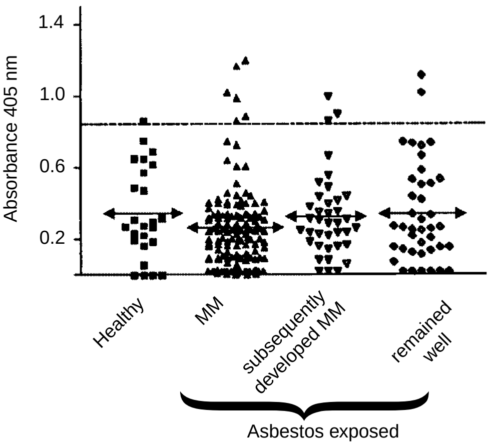

**Figure 2** Anti-p53 antibodies in MM. Serum levels of anti-p53 antibodies in healthy controls, MM patients, and individuals with a history of exposure to asbestos. Individuals exposed to asbestos were further divided into those that later went on to develop MM and those who remained disease-free at the time of this study. Presented is the mean absorbance of duplicates of a 1/50 dilution of serum measured by ELISA. The horizontal dashed line denotes an absorbance value 2 SD above the mean for the control population. The mean absorbance for each population is shown by a solid line with double headed arrows over expression with ONE58 cells having the highest levels of p53 in the MM cell lines, followed by STY51 and NO36 cells. The expression of p53 in JU77 and LO68 was lower than that of the other over-expressing cell lines but still >5 and >2-fold higher, as determined by densitometric comparison (data not shown) than that of normal human PBL.

### Anti-p53 antibodies in MM

There was substantial variation in the titre of anti-p53 antibodies in the control population of healthy individuals, with the mean relative absorbance of this population being 0.344 (±0.25). No segregation of the control population into sub-populations could be demonstrated on the basis of age, sex or smoking status (data not shown). The mean serum anti-p53 levels in 88 patients with MM, (0.244 ±0.23) was not significantly different from controls. However, 6 patients had antibody levels greater than 2 standard deviations above the mean of the control population (Figure 2). No correlation between anti-p53 antibody levels and patient survival was demonstrated. Survival of these 6 patients ranged from 3 months after diagnosis to 2.5 years after diagnosis (patient still alive).

The titre of anti-p53 antibodies was also determined in sera from people that had been exposed to asbestos, but at the time the samples were taken did not have MM. This asbestos-exposed group was further divided into those that displayed no evidence of disease at the time of the writing of this report, and those that had developed MM. The mean relative absorbance of asbestos-exposed/healthy samples was 0.321 (±0.28) and of asbestos-exposed/pre-MM samples was 0.306 (±0.23), which was not significantly different. Furthermore, there was no significant difference between the asbestos-exposed cohort and either controls or MM patients. Two individuals from the asbestos-exposed/healthy cohort and 3 individuals from the asbestos-exposed/pre-MM cohort exhibited titres above the significance line (Figure 2).

### Temporal changes in anti-p53 titre

Sera taken before the clinical diagnosis of disease were not available from the 6 MM patients with anti-p53 antibodies. However, serial sera samples were available from some individuals exposed to asbestos who then went on to develop MM. Of the 13 individuals examined, none showed a greater than 0.2 Unit change in levels of anti-p53 antibodies over a 3 to 4 year period (Figure 3A and data not shown). Similarly, the two individuals with elevated p53 antibodies (Figure 2), who were exposed to asbestos but had not, so far, developed MM showed no change in anti-p53 levels over time (Figure 3B).

Importantly, no increase of greater than 0.2 Units in anti-p53 antibody levels was demonstrated in any of 17 patients examined at various times through the progression of their disease. Where several samples were available the anti-p53 titre was seen to be remarkably consistent (Figure 3C).

### DISCUSSION

6 of 88 patients (7%) in this study with MM had elevated levels of anti-p53 antibodies present in their sera. Compared to other forms of cancer this is a low frequency (Lubin et al, 1995). However, there is a large variation in anti-p53 antibody levels reported between tumour types possibly reflecting the different properties of the malignancy which could clearly be the case for MM.

Antibodies to p53 probably arise in response to increased antigen load, secondary to the unusually large amounts of protein that accumulate because of mutations in the gene. It is clear that in general, mutations are not a pre-requisite for an autoantibody response and, indeed, there are reports in the literature of p53 antibodies in the absence of genetic mutation (Angelopoulou et al, 1994). Overexpression of translation initiation factor (eIF)-4 gamma, as a consequence of gene amplification has been shown to correlate with an immune response in patients with squamous cell lung carcinoma (Brass et al, 1999). Similarly a serologic response to HER2/neu, an oncogene amplified in certain cancers strongly supports the notion that overexpression is sufficient to initiate a humoral immune response in some individuals (Cheever et al, 1995). Such is probably the case for p53 antibodies generated in MM patients, although the p53 status of any of the patients in this study is not known. Further work is necessary to establish if there is a causal relationship between the level of expression and the elicitation of a humoral response.

 © 2001 Cancer Research Campaign

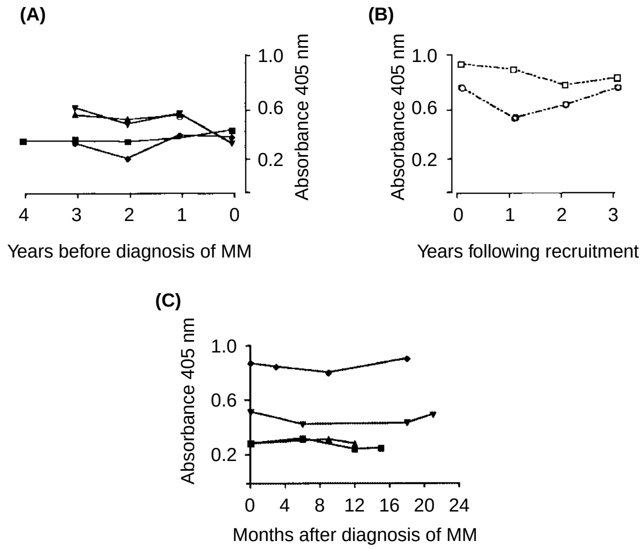

**Figure 3** Serial anti-p53 antibody measurements. anti-p53 antibodies were measured in serum from individuals exposed to asbestos who were diagnosed with MM in the year following the final sample (**A**). Similarly, antibodies were measured in serum, collected on an annual basis from two individuals exposed to asbestos that did not develop MM (**B**). Serum levels of anti-p53 antibodies were measured in four MM patients through the progression of their disease (**C**). Presented is the mean of duplicates of a 1/50 dilution measured by ELISA as Figure 2

The *p53* gene is rarely mutated in MM yet overexpression of p53 protein occurs frequently (Esposito et al, 1997; Lee and Testa, 1999; Mayall et al, 1999; and data shown here). In normal cells, p53 is present at low levels because the protein is very rapidly degraded following synthesis (Ashcroft and Vousden, 1999). Protein accumulation could result from a defect in this pathway or in one of the pathways that stabilize or activate p53. Stabilization of p53 can result following inhibition of the Mdm2-mediated degradation pathway, modulation of p53 phosphorylation, cytoplasmic sequestration, or expression of inhibitors of Mdm2 function such as $p14^{ARF}$ (Ashcroft and Vousden, 1999). In MM cell lines, Mdm2 appears to function normally (Ungar et al, 1996) and $p14^{ARF}$ is located on a chromosomal region that is frequently deleted in MM (Murthy and Testa, 1999) which suggests that p53 protein accumulation results for other reasons.

SV40 like sequences have been found in up to 60% of MM and also in several other relatively rare malignancies (Carbone et al, 1999). SV40 infection causes the in vitro transformation of normal cells. Transformation occurs predominantly through the action of the SV40 large tumour (T) antigen. T antigen has many functions including the sequestration and inactivation of the tumour suppressor gene products, p53 and Rb. Interestingly, transfection of T antigen into normal cells results in accumulation of p53 protein, this p53 is subsequently bound to T antigen in large complexes (Gonin et al, 1999). Recently, we have demonstrated the presence of SV40-like sequences in the MM cell lines examined in the current work (McLaren et al, 2000), allowing for the possibility that the p53 accumulation in these cell lines could be a result of infection with SV40 or a related virus.

p53 serum antibodies have been used as prognostic indicators in several different types of cancer. Because of the lack of treatment options for MM and the short mean survival of 9 months the usefulness of anti-p53 antibodies in MM appears limited. In the present study, only 6 of the 88 patients sera examined had elevated p53 antibodies and there was no correlation between antibody levels and survival.

One of the major objectives of this study was to determine the utility of anti-p53 antibodies as diagnostic or prognostic surrogate markers of MM. A cheap and reliable test for MM would have immense value for those people in the 'at-risk' population by virtue of their prolonged exposure to asbestos. Given the poor prognosis of individuals with MM it may prove useful if treatment options could be explored earlier. Also, given the experimental nature of newer therapeutic options, markers of disease progression would be an invaluable adjunct to clinical trials. However, the present study showed no difference between individuals that had been exposed to asbestos but were segregated upon the basis of whether or not they went on to develop MM. And furthermore, there was no increase in p53 antibody levels over time in these populations. Thus anti-p53 antibody levels do not serve as a diagnostic indicator for MM.

The clinical relevance of antibodies against p53 appears to be dependent on the tumour type. In the case of MM, anti-p53 antibodies are observed only at a low frequency in the sera of patients and their presence does not serve as either a prognostic or diagnostic indicator.

### ACKNOWLEDGEMENT

RAL is the recipient of a James Hardie Industries Molecular Biology Fellowship.

### REFERENCES

Andersson M, Wallin H, Jonsson M, Nielsen LL, Visfeldt J, Vyberg M, Bennett WP, De Benedetti VM, Travis LB and Storm HH (1995) Lung carcinoma and malignant mesothelioma in patients exposed to Thorotrast: incidence, histology and p53 status. *Int J Cancer* **63**: 330–336

Angelopoulou K, Diamandis EP, Sutherland DJA, Kellen JA and Bunting PS (1994) Prevalence of serum antibodies against the p53 tumor suppressor gene protein in various cancers. *Int J Cancer* **58**: 480–487

Ashcroft M and Vousden KH (1999) Regulation of p53 stability [Review]. *Oncogene* **18**: 7637–7643

Brass N, Racz A, Bauer C, Heckel D, Sybrecht G and Meese E (1999) Role of amplified genes in the production of autoantibodies. *Blood* **93**: 2158–2166

Carbone M, Fisher S, Powers A, Pass HI and Rizzo P (1999) New molecular and epidemiological issues in mesothelioma: Role of SV40. *J Cell Physiol* **180**: 167–172

Cheever MA, Disis ML, Bernhard H, Gralow JR, Hand SL, Huseby ES, Qin HL, Takahashi M and Chen W (1995) Immunity to oncogenic proteins [Review]. *Immunological Reviews* **145**: 33–59

Davidson JA, Musk AW, Wood BR, Morey S, Ilton M, Yu LL, Drury P, Shilkin K and Robinson BWS (1998) Intralesional cytokine therapy in cancer – a pilot study of GM-CSF infusion in mesothelioma. *Journal of Immunotherapy* **21**: 389–398

de Klerk N, Musk AW, Ambrosini GL, Eccles JL, Hansen J, Olsen N, Watts VL, Lund HG, Pang SC, Beilby J and Hobbs MS (1998) Vitamin A and cancer prevention II: comparison of the effects of retinol and beta-carotene. *Int J Cancer* **75**: 362–7

Esposito V, Baldi A, De LA, Claudio PP, Signoriello G, Bolognese A, Centonze P, Giordano GG, Caputi M, Baldi F and Giordano A (1997) p53 immunostaining in differential diagnosis of pleural mesothelial proliferations. *Anticancer Res* **17**: 733–736

Gadducci A, Ferdeghini M, Buttitta F, Cosio S, Fanucchi A, Annicchiarico C, Gagetti O, Bevilacqua G and Genazzani AR (1999) Assessment of the

© 2001 Cancer Research Campaign prognostic relevance of serum anti-p53 antibodies in epithelial ovarian cancer. *Gynecologic Oncology* **72**: 76–81

Gansauge S, Gansauge F, Negri G, Galle P, Muller J, Nussler AK, Poch B and Beger HG (1996) The role of anti-p53-autoantibodies in pancreatic disorders. *Int J Pancreatol* **19**: 171–178

Gonin S, Diaz-Latoud C, Richard MJ, Ursini MV, Imbo A, Manero F and Arrigo AP (1999) p53/T-antigen complex disruption in T-antigen transformed NIH3T3 fibroblasts exposed to oxidative stress: correlation with the appearance of a Fas/APO-1/CD95 dependent, caspase independent, necrotic pathway. *Oncogene* **18**: 8011–8023

Gottschlich S, Folz BJ, Goeroegh T, Lippert BM, Maass JD and Werner JA (1999) A new prognostic indicator for head and neck cancer – p53 serum antibodies? *Anticancer Research* **19**: 2703–2705

Hammel P, Leroy-Viard K, Chaumette MT, Villaudy J, Falzone MC, Rouillard D, Hamelin R, Boissier B and Remvikos Y (1999) Correlations between p53-protein accumulation, serum antibodies and gene mutation in colorectal cancer. *Int J Cancer* **81**: 712–718

Lai CL, Tsai CM, Tsai TT, Kuo BIT, Chang KT, Fu HT, Perng RP and Chen JY (1998) Presence of serum anti-p53 antibodies is associated with pleural effusion and poor prognosis in lung cancer patients. *Clin Cancer Res* **4**: 3025–3030

Lang C, Unteregger G, Kartarius S, Gunther J, Bonkhoff H, Montenarh M and Zwergel T (1998) p53 autoantibodies in patients with urological tumours. *Brit J Urol* **82**: 721–725

Lechner JF, Tesfaigzi J and Gerwin BI (1997) Oncogenes and tumor-suppressor genes in mesothelioma – a synopsis. *Environ Health Perspect* **105**: 1061–1067

Lee WC and Testa JR (1999) Somatic genetic alterations in human malignant mesothelioma (Review). *Int J Oncol* **14**: 181–188

Lenner P, Wiklund F, Emdin SO, Arnerlov C, Eklund C, Hallmans G, Zentgraf H and Dillner J (1999) Serum antibodies against p53 in relation to cancer risk and prognosis in breast cancer: a population-based epidemiological study. *Brit J Cancer* **79**: 927–932

Levine AJ (1997) p53, the cellular gatekeeeper for cell growth and division. *Cell* **88**: 323–331

Lubin R, Schlichtholz B, Teillaud JL, Garay E, Bussel A, Wild CP and Soussi T (1995) p53 antibodies in patients with various types of cancer – assay, identification, and characterization. *Clini Cancer Res* **1**: 1463–1469

Manning LS, Whitaker D, Murch AR, Garlepp MJ, Davis MR, Musk AW and Robinson BW (1991) Establishment and characterization of five human malignant mesothelioma cell lines derived from pleural effusions. *Int J Cancer* **47**: 285–290

Mayall FG, Jacobson G and Wilkins R (1999) Mutations of p53 gene and SV40 sequences in asbestos associated and non-asbestos-associated mesotheliomas. *J Clin Pathol* **52**: 291–293

McLaren BR, Haenel T, Stevenson S, Mukherjee S, Robinson BWS and Lake RA (2000) SV40 like sequences in cell lines and tumour biopsies from Australian malignant mesotheliomas. *Aust NZJ Med* **30**: 450–456

Metcalf RA, Welsh JA, Bennett WP, Seddon MB, Lehman TA, Pelin K, Linnainmaa K, Tammilehto L, Mattson K and Gerwin BI (1992) p53 and Kirstenras mutations in human mesothelioma cell lines. *Cancer Res* **52**: 2610–2615

Mitsudomi T, Suzuki S, Yatabe Y, Nishio M, Kuwabarn M, Gotoh K, Hatooka S, Shinoda M, Suyama M, Ogawa M, Takahashi T and Ariyoshi Y (1998) Clinical implications of p53 autoantibodies in the sera of patients with non-small-cell lung cancer. *J Natl Cancer Inst* **90**: 1563–1568

Mor O, Yaron P, Huszar M, Yellin A, Jakobivitz O, Broksimoni F, Rechavi G and Reichert N (1997) Absence of p53 mutations in malignant mesotheliomas. *Am J Resp Cell Mol Biol* **16**: 9–13

Murthy SS and Testa JR (1999) Asbestos, chromosomal deletions, and tumor suppressor gene alterations in human malignant mesothelioma. *J Cell Physiol* **180**: 150–157

Musk AW, Bowman RV, Christmas TI and Robinson BW (1992) Management of malignant mesothelioma. In: Henderson DW, Shikin KB, Langlois SW and Whitaker D (eds) *Malignant Mesothelioma.* Hemisphere Publishing Corporation: New York. pp. 293–302

Musk AW, de KN, Ambrosini GL, Eccles JL, Hansen J, Olsen NJ, Watts VL, Lund HG, Pang SC, Beilby J and Hobbs MS (1998) Vitamin A and cancer prevention I: observations in workers previously exposed to asbestos at Wittenoom, Western Australia. *Int J Cancer* **75**: 355–361

Nakajima K, Suzuki T, Shimada H, Hayashi H, Takeda A and Ochiai T (1999) Detection of preoperative serum anti-p53 antibodies in gastric cancer. *Tumor Biology* **20**: 147–152

Ramael M, Lemmens G, Eerdekens C, Buysse C, Deblier I, Jacobs W and van Marck E (1992) Immunoreactivity for p53 protein in malignant mesothelioma and non-neoplastic mesothelium. *J Pathol* **168**: 371–375

Robinson C, Robinson BWS and Lake RA (1998) Sera from patients with malignant mesothelioma can contain autoantibodies. *Lung Cancer* **20**: 175–184

Robinson C, Callow M, Stevenson S, Scott B, Robinson BW and Lake RA (2000) Serologic responses in patients with malignant mesothelioma: evidence for both public and private specificities. *Am J Respir Cell Mol Biol* **22**: 550–556

Saffroy R, Lelong JC, Azoulay D, Salvucci M, Reynes M, Bismuth H, Debuire B and Lemoine A (1999) Clinical significance of circulating anti-p53 antibodies in European patients with hepatocellular carcinoma. *British Journal of Cancer* **79**: 604–610

Schneider J, Presek P, Braun A, Bauer P, Konietzko N, Wiesner B and Woitowitz HJ (1999) p53 protein, EGF receptor, and anti-p53 antibodies in serum from patients with occupationally derived lung cancer. *British Journal of Cancer* **80**: 1987–1994

Ungar S, Van de Meeren A, Tammilehto L, Linnainmaa K, Mattson K and Gerwin BI (1996) High levels of MDM2 are not correlated with the presence of wild-type p53 in human malignant mesothelioma cell lines. *Br J Cancer* **74**: 1534–1540

Upham J, Musk A, van Hazel G, Byrne M and Robinson B (1993) Interferon alpha and doxorubicin in malignant mesothelioma. *Aust NZ J Med* **23**: 683–687

Vogl FD, Stickeler E, Weyermann M, Kohler T, Grill HJ, Negri G, Kreienberg R and Runnebaum IB (1999) p53 autoantibodies in patients with primary ovarian cancer are associated with higher age, advanced stage and a higher proportion of p53-positive tumor cells. *Oncology* **57**: 324–329

Wu CW, Lin YY, Chen GD, Chi CW, Carbone DP and Chen JY (1999) Serum anti-p53 antibodies in gastric adenocarcinoma patients are associated with poor prognosis, lymph node metastasis and poorly differentiated nuclear grade. *British Journal of Cancer* **80**: 483–488

*© 2001 Cancer Research Campaign*
